# Blame—a novel by Tony Holtzman

**DOI:** 10.1007/s12687-017-0334-4

**Published:** 2017-09-14

**Authors:** Martina C. Cornel

**Affiliations:** Department of Clinical Genetics, Section Community Genetics, Amsterdam Public Health research institute, VU University Medical Center, BS7 A509, PO Box 7057, 1007 MB Amsterdam, The Netherlands

## Abstract

After writing many scientific articles at the interface of genetics and society, Neil A (Tony) Holtzman published a novel in Autumn 2016: *Blame*. This book review summarizes several of the story lines, some of which are related to the Inclusion of Diverse Populations in Genomics Research and Health Services, the topic of a special issue of the *Journal of Community Genetics*.

Neil A (Tony) Holtzman has a long-lasting interest in Community Genetics. PubMed includes his scientific contributions on ethical aspects of genetic testing, patents, the quality of media reports on discoveries related to human genetic diseases, genetic susceptibility testing for Alzheimer’s disease, among others. The most recent publication of Tony Holtzman, released in late 2016, however is a novel: *Blame*. A fascinating story may not be completely true but still reminds the reader of many realities of genetic research. If you want to discover the story lines on your own, I advise you not to continue reading this book review as it might spoil your reading experience. This book review does talk about key themes and parts of the plot, as well as some of the questions, this novel stresses about translational genomics research.

## Public or private

Once the bright young star on the medical school faculty, Jason Pearce finds himself with no funding from the NIH for his mouse studies on Alzheimer’s disease, and he is forced to accept funding from the commercial company Ventures Unlimited, Ltd. Faculty-industry relations are regulated to make commercially supported research possible to a limited extent—but what does this do to the integrity of the young star once he gets older? The celebrity can now afford to drive a Lamborghini, a sports car that is more expensive than the other faculty members can afford. As a reward for his scientific work, he builds a house on an enormous property, with lots of luxury planned for. Will he remember the principles that he used to discuss with Professor Chapman, author of *Patents and Profits*—*The Demise of the University*, on skepticism about the practical applications of one’s own work? And what will happen to the University that agreed to generate income with fees from licensing and royalties to fill the void when biotechnology increasingly held promises but federal grants were not keeping up with this explosion of research?

## Mice or men

In experiments in mice with one deafness allele, they appear to be more forgetful than wildtype mice. Therefore, they can serve as a model for Alzheimer’s disease, the deafness gene now being called a memory gene. The injection of the gene in mice brain is successful in treating and preventing Alzheimer’s disease. A clinical trial in people at risk of Alzheimer’s disease is set up. What would be the difference between animal experiments or human studies in terms of competences needed for the principal investigator and his responsibilities? With high expectations of potential use in many people around the world, the venture capital company is happy to help the project to move forward.

## White or black

The clinical trial takes place in a city with a high proportion of people of African ancestry. One third of the population in the city limits is African-American, but none of the doctors caring for the study participants in the trial is African-American. After the first adverse event is reported, it turns out that genetic risk profiles for black and white are not the same for Alzheimer’s disease. In fact, the African-American woman that is fatally harmed should not have been included in the trial, as is evident in retrospect. Preventable harm has been done to research subjects, especially to a population that is underrepresented among students and staff in the Hospital and University, but overrepresented in the dangerous trial. A reverend resembling Martin Luther King Jr. stands up to voice the rights of his black community. The story of *The Immortal Life of Henrietta Lacks* comes to the mind of the reader as well—who suffers and who profits when it comes to science?

## Who is to blame?

After the adverse event, the question surfaces “who is to blame?” The laboratory scientist who moved from laboratory science to a clinical trial? The commercial company that wanted to sponsor Alzheimer’s trials as soon as possible? The university that accepted the terms of collaboration, including sharing patents and conditions for publication? The Institutional Review Board that approved the clinical trial, without asking some relevant questions? The clinician who treated the patients in the trial? If ancestral diversity was not taken into account sufficiently, are they all to blame?

## The role of media reports

In several phases of his career, Jason Pearce’s activities are covered by journalists. The success story on his mice experiments raises hope in families of Alzheimer’s patients. The adverse event attracts attention of an investigative journalist, who asks the questions that were somehow overlooked by the researchers. Do we recognize an interesting parallel with Tony Holtzman who reflects on everything he experienced in genetic science—and thus makes us reflect on biotechnology and society?
